# Clinical Management of COVID-19: A Review of Pharmacological Treatment Options

**DOI:** 10.3390/ph14060520

**Published:** 2021-05-28

**Authors:** Ashli M. Heustess, Melissa A. Allard, Dorothea K. Thompson, Pius S. Fasinu

**Affiliations:** 1 School of Pharmacy, College of Pharmacy and Health Sciences, Campbell University, Buies Creek, NC 27501, USA; amheustess0305@email.campbell.edu (A.M.H.); maallard1025@email.campbell.edu (M.A.A.); 2 Department of Pharmaceutical Sciences, College of Pharmacy and Health Sciences, Campbell University, Buies Creek, NC 27501, USA; dthompson@campbell.edu

**Keywords:** COVID-19, SARS-CoV-2, antivirals, casirivimab/imdevimab, convalescent plasma, remdesivir

## Abstract

Since the outbreak and subsequent declaration of COVID-19 as a global pandemic in March 2020, concerted efforts have been applied by the scientific community to curtail the spread of the disease and find a cure. While vaccines constitute a vital part of the public health strategy to reduce the burden of COVID-19, the management of this disease will continue to rely heavily on pharmacotherapy. This study aims to provide an updated review of pharmacological agents that have been developed and/or repurposed for the treatment of COVID-19. To this end, a comprehensive literature search was conducted using the PubMed, Google Scholar, and LitCovid databases. Relevant clinical studies on drugs used in the management of COVID-19 were identified and evaluated in terms of evidence of efficacy and safety. To date, the FDA has approved three therapies for the treatment of COVID-19 Emergency Use Authorization: convalescent plasma, remdesivir, and casirivimab/imdevimab (REGN-COV2). Drugs such as lopinavir/ritonavir, umifenovir, favipiravir, anakinra, chloroquine, hydroxychloroquine, tocilizumab, interferons, tissue plasminogen activator, intravenous immunoglobulins, and nafamosat have been used off-label with mixed therapeutic results. Adjunctive administration of corticosteroids is also very common. The clinical experience with these approved and repurposed drugs is limited, and data on efficacy for the new indication are not strong. Overall, the response of the global scientific community to the COVID-19 pandemic has been impressive, as evident from the volume of scientific literature elucidating the molecular biology and pathophysiology of SARS-CoV-2 and the approval of three new drugs for clinical management. Reviewed studies have shown mixed data on efficacy and safety of the currently utilized drugs. The lack of standard treatment for COVID-19 has made it difficult to interpret results from most of the published studies due to the risk of attribution error. The long-term effects of drugs can only be assessed after several years of clinical experience; therefore, the efficacy and safety of current COVID-19 therapeutics should continue to be rigorously monitored as part of post-marketing studies.

## 1. Introduction

Severe acute respiratory syndrome coronavirus 2 (SARS-CoV-2) was declared the infectious agent of the global pandemic coronavirus disease 2019 (COVID-19) by the World Health Organization (WHO) on 11 March 2020 [1]. Originating from its epicenter in Wuhan, China, SARS-CoV-2 quickly spread to other countries, with the first U.S. case reported in January 2020 in Washington state. At the time of this authorship, more than 166 million confirmed cases worldwide and over 3.4 million deaths in 222 countries have been attributed to COVID-19 [2]. In the absence of gold standard treatments, the disease can progress rapidly and lead to pneumonia, resulting in long-term organ damage and even death in some patients.

SARS-CoV-2 represents the third occurrence of a zoonotic virus within the coronavirus family to cause severe disease in human hosts lacking pre-existing immunity. Prior to the SARS-CoV-2 pandemic, humans have endured two outbreaks in the past two decades—SARS-CoV in 2003, and Middle East respiratory syndrome coronavirus (MERS-CoV)—caused by newly emerged novel coronaviruses that crossed species barriers [3]. Coronaviruses of the family *Coronaviridae* are large, enveloped, single-stranded RNA viruses found in both humans and various animal species. Their distinctive ‘corona’ or crown-like morphology visualized by electron microscopy is due to the presence of spike-like glycoproteins emanating from the surface of the viral envelope [4,5].

Coronaviruses are broadly categorized into four genera: alpha-CoV, beta-CoV, gamma-CoV, and delta-CoV [6,7]. As with MERS-CoV and SARS-CoV, SARS-CoV-2 is classified as a beta-CoV and is characterized by viral genetic diversity, genomic plasticity, and multiple host adaptability due to high mutation rates [8]. Coronaviruses belonging to the alpha-CoV and beta-CoV genera are transmissible to humans, and genome similarity evidence indicates that these viruses originated from bats, although the pangolin has been suggested to be an intermediate host for human infection by SARS-CoV-2 [9,10,11,12,13,14].

The primary mode of SARS-CoV-2 transmission is through respiratory droplets expelled during face-to-face exposure, although spread via contact with contaminated surfaces is also possible [15]. Infectivity is dependent upon three structural proteins (E, M, and S) in the viral envelope that have critical functions in the replication cycle of SARS-CoV-2 [16]. Envelope (E) proteins form viroporins, or ion channels, in the lipid bilayer and are important for viral maturation [17]. Membrane (M) proteins play essential roles in the morphogenesis and assembly of new SARS-CoV-2 progeny by interacting with other structural proteins [18]. The spike (S) is a surface-exposed glycoprotein that is essential for SARS-CoV-2 attachment, fusion, and entry into the host cell [19]. Infection is initiated by binding of the spike glycoprotein to the human angiotensin-converting enzyme 2 (hACE2) receptor on SARS-CoV-2 target cells, such as nasal and bronchial epithelial cells and pneumocytes [20]. The dissemination of SARS-CoV-2 infection to extra-pulmonary sites in COVID-19 patients is due to the wide cellular distribution of the hACE2 receptor, which is also found on tissues of the gastrointestinal tract, cardiovascular, urogenital, and central nervous systems [21]. Cleavage of the S protein by the host cell-associated transmembrane protease serine 2 (TMPRSS2) activates the S2 domain for fusion of the viral envelope with the cell membrane, permitting entry of the viral nucleocapsid [20].

SARS-CoV-2 infection is characterized by variable clinical severity. The clinical picture of COVID-19 ranges widely from asymptomatic or mild cold-like symptoms to acute respiratory distress syndrome (ARDS), respiratory failure, multiple organ failure, and death. The most common presenting features in adults are fever (up to 90%), dry cough (60–86%), shortness of breath (53–80%), fatigue (38%), myalgia (15–44%), sputum production (33%), sore throat (13.9%), and headache (13.6%) [22,23,24,25]. Nonclassical gastrointestinal symptoms such as diarrhea (3.8%) and vomiting (5.0%) occur infrequently [26]. In addition, a majority of reported COVID-19 cases (64–80%) also presented with ageusia and anosmia [27,28]. Clinical evidence suggests that dysregulated and excessive proinflammatory cytokine release (a ‘cytokine storm’) constitutes a major cause of ARDS and is associated with severe health deterioration in critically ill COVID-19 patients (reviewed in Ye et al., 2020 [29]). A poor prognosis from COVID-19 is disproportionately higher among individuals of advanced age and who have pre-existing chronic medical conditions. Hospital mortality is less than 5% for COVID-19 patients who are younger than 40 years, while the hospital death rate rises substantially to 35% for 70- to 79-year-old patients, and to >60% for 80- to 89-year-old patients [23]. The potential long-term health impacts in survivors of severe COVID-19 currently remain unknown.

Therapeutics for the prevention and management of SARS-CoV-2 infection have changed dramatically since the early progression of the pandemic. As of the completion of this study, three vaccines for the prevention of COVID-19 have received approval by the U.S. Food and Drug Administration (FDA) under Emergency Use Authorization (EUA) and are currently being administered to the public: two mRNA vaccines (Moderna mRNA-1273 and the Pfizer–BioNTech BNT162b2), and the Janssen viral vector vaccine [30]. The Oxford-AstraZeneca prophylactic (AZD1222), an adenoviral vector-based vaccine, is being used in the United Kingdom. While vaccines constitute a critical part of the public health strategy to reduce disease burden, the management of the disease will continue to rely heavily on pharmacotherapy. Therefore, the aim of this paper is to provide an updated overview of pharmacological agents that have been repurposed, evaluated, or developed for COVID-19 management, including a review of published data for evidence (or lack thereof) of efficacy and safety with regard to identified agents.

## 2. Methodology

A comprehensive literature search was conducted on September 16, 2020, using the PubMed, Google Scholar and LitCovid databases. An updated search was conducted monthly until February 2021. Our search focused on the identification of the most commonly used drugs in COVID-19 treatment. Included articles were restricted to clinical studies involving clinical trials, case reports, and case series. Search terms were predefined and consisted of the combination of the following: “COVID”, “coronavirus”, “treatments”, “drugs”, and “drug therapy”. Based on the literature search results, the most common therapies were chloroquine, hydroxychloroquine, anakinra, interferons, corticosteroids (dexamethasone and methylprednisolone), nafamostat, arbidol, lopinavir/ritonavir, remdesivir, tocilizumab, convalescent plasma, and combinations thereof. Further searches were conducted to include these drugs as search terms, in relation to COVID-19 treatment. Articles reviewed were limited to those available in the English language.

## 3. Results

**Figure 1:**
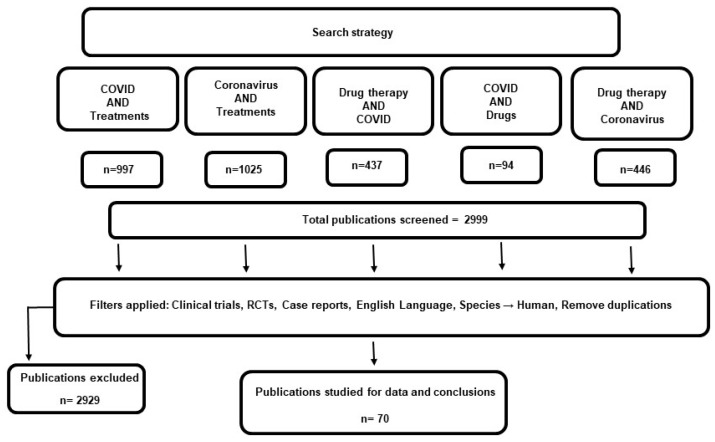
Search results and study selection.

**Figure 2:**
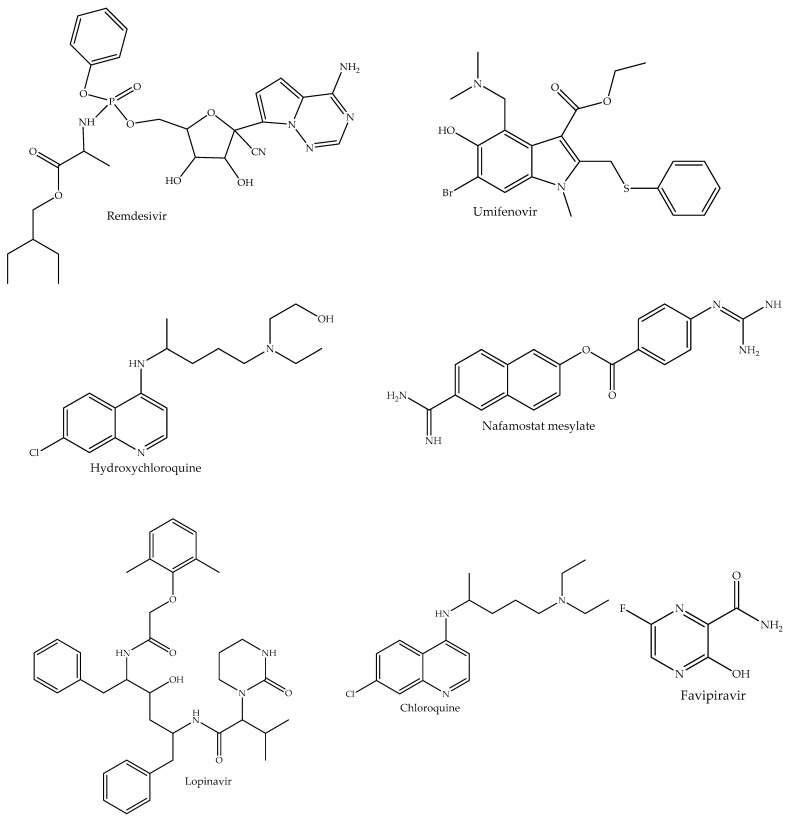
Chemical structures of small-molecule drugs that have been approved/repurposed for the treatment of COVID-19.

Since the discovery of SARS-Cov-2 and the declaration of COVID-19 as a global pandemic, numerous studies have evaluated different pharmacological agents against the novel virus. Figure 1 summarizes the study selection process and the search results. The initial search returned a total of 2999 articles which were screened for inclusion criteria. A total of 70 articles were reviewed in this study. In Table 1, a summary of the clinical findings on the effectiveness of current therapeutics for COVID-19 is presented. The approved and repurposed drugs are from different chemical and pharmacological classes, reflecting the diversity of the mechanisms of action and potential therapeutic benefits (Figure 2).

### 3.1. Approved Therapeutics

To date, the FDA has approved three therapies for the treatment of COVID-19 under EUA: convalescent plasma, remdesivir, and casirivimab/imdevimab (regeneron). Several drugs, including antiviral and antiparasitic agents, have been repurposed for the treatment of COVID-19. Supportive therapies including immunosuppressants have also been used.

#### 3.1.1. Convalescent Plasma

Convalescent plasma (CP), obtained from recovering patients, provides passive immunity in actively infected patients. CP has been utilized in previous viral pandemics. For example, CP demonstrated efficacy and superiority over placebo (58.3% vs. 15.6%, *p* < 0.001) in an earlier study with patients suffering from SARS-CoV infection [54]. CP has also been used for the prevention and/or treatment of such viral infections as poliomyelitis [55], measles [56], and mumps [57]. Case series suggested the efficacy of CP in the treatment of Machupo virus associated with Bolivian hemorrhagic fever [58], Lassa fever in Nigeria [59], Junin virus associated with Argentinian hemorrhagic fever [60], and Ebola viral infection [61]. Use was safe and effective against influenza [62], influenza A (H5N1) infection [63], and H1N1 pandemic influenza [64].

On 23 August 2020, the FDA approved an EUA for CP to treat hospitalized patients with COVID-19. Compared with other blood products and therapeutics for COVID-19, CP had a clear scientific basis for use. For severely ill COVID-19 patients, CP appeared to be a potential therapy with no serious adverse effects reported [50,52,65]. However, concerns persist regarding dosing and the need for standardization due to varying donor titer amounts [49,50,52].

Cases and case reports have highlighted the utility and efficacy of CP in the treatment of COVID-19 (Table 1). Clinical improvement has been reported for patients who were immunocompromised at the time of contracting SARS-CoV-2 [46], patients who were refractory to antiviral and supportive care [44], and a pediatric patient with severe COVID-19 whose previous treatment with antiviral drugs and immune modulators failed [40]. As a biologic, concerns about safety across patient populations have been raised. However, CP has been well tolerated based on several published clinical reports (Table 1). Jafari et al. [39] reported the use of CP in a 26-year-old COVID-19 patient who was pregnant with twins. CP was infused following caesarian section, and treatment failure with meropenem, azithromycin, and hydroxychloroquine. Given on the sixth day of hospitalization, CP administered with favipiravir was associated with improved clinical outcomes and the patient was discharged two weeks after admission. However, efficacy across all patients has not been established for CP.

In an open-label, multi-center, randomized controlled trial involving 103 COVID-19 patients with severe or life-threatening symptoms, CP used as adjunctive therapy did not cause any significant difference in 28-day mortality compared to standard therapy [53]. Difference in time spent before discharge was also not significant. The study was terminated early, without reaching the planned 200 recruits. Overall, although CP may offer clinical benefits in COVID-19 treatment, current data are mixed and largely inconclusive. Published case reports and case series often combine CP use with other therapies, making it difficult to attribute efficacy to CP. More controlled studies are required to establish the efficacy of CP in COVID-19 treatment.

#### 3.1.2. Remdesivir

Remdesivir is a monophosphoramide nucleoside analog with broad-spectrum antiviral activity. Once activated by host cellular kinases, the active remdesivir triphosphate exerts antiviral activity by inhibiting the viral RNA-dependent RNA polymerase, competing with adenosine triphosphate for incorporation into the viral RNA and terminating viral RNA synthesis. Prior to the current pandemic, in vitro studies demonstrated that remdesivir exhibits strong antiviral activity [66]. Previously developed to treat Ebola virus infection [67,68], remdesivir has selective cytotoxic activity against SARS-CoV-2 [69]. Other preclinical animal studies have shown that remdesivir reduces viral load in lung tissues, while enhancing pulmonary functions [70].

Remdesivir was one of the drugs used to treat the first case of COVID-19 in the United States, with remarkable results [71]. Since then, there have been numerous case reports on the use of remdesivir in COVID-19 with documented improvements in viral loads and symptomatology (Table 1). Particularly noteworthy was the use of remdesivir in a pregnant woman in her third trimester, resulting in significant clinical response and patient home discharge, without requiring emergency delivery [34]. Another important case report involved an immunocompromised patient who received a two 10-day courses of remdesivir starting at day 24 and day 45 after the onset of symptoms [36]. Not only did symptoms improve, but the patient subsequently tested negative for the virus after some episodes of relapse. These studies demonstrated the safe and effective use of remdesivir in pregnant and immunosuppressed patients.

In a prospective open-label study, remdesivir was effective in treating COVID-19 pneumonia in patients with severe symptoms [32]. This study, which enrolled 35 critically ill COVID-19 patients (18 ICU and 17 infectious disease ward (IDW) patients), recorded significant clinical responses following remdesivir use such that 6 and 14 patients had been discharged from the ICU and IDW, respectively, by day 28. Another clinical trial that evaluated the efficacy of remdesivir examined 5- and 10-day courses in a population of 397 randomized COVID-19 patients. In this study, 200 and 197 patients received intravenous remdesivir for 5 or 10 days, respectively (200 mg on day 1 and subsequently 100 mg daily) with both groups showing similar levels of clinical improvements by day 14 [38].

Two major randomized, double-blind, placebo-controlled, multicenter clinical trials have been reported for remdesivir. In the first report by Wang et al. [47], a total of 237 patients were enrolled, with 158 and 79 randomized to the remdesivir and placebo groups, respectively. Patients were allowed concurrent use of other antivirals and corticosteroids. Although patients taking remdesivir had faster clinical improvement, there was no statistically significant difference in the time to clinical improvement in the two groups. In the trial conducted by Beigel et al. [37], 1062 patients were randomized, with 541 receiving remdesivir and 521 receiving the placebo. Median recovery time with remdesivir was 10 days compared to 15 days for the placebo group. A summary of clinical experience with remdesivir is provided in Table 1. Although responses to remdesivir have been mixed, the drug appears to be safe with overall clinical benefit sometimes attributable to co-treatment with other therapies. Approved previously for use in Japan, Taiwan, India, Singapore, and the United Arab Emirates, remdesivir received a one-year conditional marketing authorization from the European Commission in July 2020. In October 2020, remdesivir was approved by the FDA for use in adult and pediatric patients (≥12 years of age and weighing at least 40 kg) for the treatment of COVID-19 requiring hospitalization.

#### 3.1.3. Casirivimab/Imdevimab

The casirivimab/imdevimab combination (REGN-COV2) was granted an EUA for outpatient use in November 2020. REGN-COV2 combines two recombinant human monoclonal antibodies (mAb) which were initially reported as a new antibody cocktail capable of targeting the spike protein of SARS-CoV-2 and withstanding antibody resistance [72]. The two mAbs were further characterized to be potent, with strong affinity for the receptor-binding domain of the spike protein [73]. In two animal models—rhesus macaque and golden hamster for mild and severe COVID-19, respectively—prophylactic casirivimab/imdevimab reduced viral load in the respiratory tract and limited disease progression, indicating therapeutic potential of REGN-COV2 in humans [74].

Analysis of casirivimab/imdevimab revealed a greater reduction in viral load for patients whose immune response had not yet started or who had a viral load at their baseline visit [15]. This longitudinal study, which analyzed 275 patients randomized equally into three groups (placebo and 2.4 g or 8.0 g of casirivimab/imdevimab), monitored the immune response to the virus. Safety profiles were similar in tests and placebo. Clinical experience with casirivimab/imdevimab is limited, and published clinical studies are sparse.

### 3.2. Repurposed and Off-Label Anti-Infective Drugs

#### 3.2.1. Lopinavir–Ritonavir

Lopinavir–ritonavir (LPV/r) is a common antiretroviral combination which acts against the viral protease enzyme and prevents the cleavage of the precursor polyprotein at the late stage of viral replication. Ritonavir is used in the LPV/r fixed combination as a potent inhibitor of the efflux p-glycoprotein and cytochrome P450 3A4 enzymes, thus enhancing the overall plasma exposure to LPV. During the 2003 SARS outbreak, LPV/r was one of the therapeutic agents explored as treatment and showed some in vitro activity against strains of SARS-CoV [75]. The authors at the time also compared the clinical efficacy of ribavirin–lopinavir/r combination (41 patients) with ribavirin alone (111 patients) against SARS-CoV and reported a better outcome with LPV/r use. Specifically, LPV/r significantly reduced the incidence of acute disease and death by day 21. Use of LPV/r was also associated with decreased intubation rate and mortality in a multicenter study of SARS-CoV [76]. This, and other limited evidence of LPV/r efficacy in combating SARS, spurred interest in exploring LPV/r for the treatment of COVID-19.

Several case reports and case series on the use of LPV/r show mixed results on its efficacy in COVID-19 treatment. While the reported clinical improvement and negative conversion in several patients were partially attributed to LPV/r, a number of the published cases showed otherwise. Two published clinical trials also showed mixed data. The study by Ye et al. [77] associated a faster clinical response and shorter course of the disease to LPV/r use. This open-label study assigned 47 hospitalized COVID-19 patients to the group receiving either the standard COVID-19 care alone (control) or the group receiving LPV/r in addition to the standard care. The control group was given interferon inhalation and umifenovir, while antibiotics and other remedies were used when indicated. The small sample size and the influence of as-needed antibiotics and multicomponent adjuvant treatment likely limited the conclusions that could be drawn from this study. In a second clinical study by Cao et al. [78], LPV/r neither shortened the time to clinical improvement nor reduced mortality at 28 days. A total of 199 patients who were hospitalized for severe COVID-19 were randomized for a 14-day treatment with either LPV/r plus the standard care (99 patients) or standard care alone (100 patients). Virological response was similar in both groups, suggesting that LPV/r may not be more effective for viral cure. Overall, LPV/r lacks convincing data to support widespread use in the treatment of COVID-19. More robust and varied clinical studies are needed to generate conclusive data.

#### 3.2.2. Umifenovir

Umifenovir (Arbidol) is an indole carboxylic acid derivative with broad-spectrum antiviral activity and is currently approved in China and Russia for the prevention and treatment of infections caused by influenza type A and B viruses [79]. It acts by inhibiting the fusion of the virion with the host cell membrane. In vitro studies have shown umifenovir to be active against a wide spectrum of viruses, including human herpesvirus, hepatitis C virus, and Ebola virus [80]. Umifenovir also has demonstrated in vitro activity against SARS-CoV and SARS-CoV-2, engendering scientific interest in this drug as a potential treatment modality for COVID-19 [81,82].

The limited clinical data that exist on the use of umifenovir in COVID-19 treatment is mixed. In a retrospective study of 33 patients who were treated with either the umifenovir–LPV combination (16 patients) or LPV/r only (17 patients), the use of umifenovir appeared to have produced a significant clinical response. By day 7 of treatment, negative conversion occurred in 75% of patients in the combination group compared to 35% in the LPV/r group, with the numbers increasing to 94% and 53%, respectively, after 14 days. Additionally, chest CT scan improvement was better with the combination therapy [83]. In another retrospective analysis of COVID-19 patients treated with LPV/r (34 cases) vs. umifenovir (16 cases), viral cure at day 14 was 56% and 100%, respectively, although disease progression was halted in both groups [84]. A third study that showed potential efficacy of umifenovir was an analysis of 62 hospitalized COVID-19 patients who received adjuvant treatment alone (20 patients) vs. umifenovir (42 patients). The results showed that the use of umifenovir was associated with a shorter course of disease and reduced the duration of hospitalization [85].

Not all studies have shown promising results for umifenovir. Two other retrospective analyses concluded that umifenovir was not associated with better viral cure or any other clinical outcome in COVID-19 treatment [86,87]. In a more elaborate randomized open-label controlled multicenter trial, 240 patients were randomized (1:1) to receive conventional COVID-19 therapy along with either umifenovir or favipiravir. Clinical recovery by day 7 was not significantly different between the groups, but umifenovir was associated with a slower rate of clinical recovery and symptom improvement [88]. With conflicting findings from these limited studies, data from multiple ongoing studies will hopefully provide more definitive conclusions with regard to the efficacy of umifenovir for COVID-19 treatment.

#### 3.2.3. Favipiravir

Favipiravir (FPV) is a broad-spectrum antiviral agent for the treatment of SARS and MERS. It is a purine analog prodrug which is phosphorylated in situ and competitively inhibits the viral RNA-dependent RNA polymerase. FPV was reported to be effective in the treatment of Ebola virus infection in mice [89]. A recent study also demonstrated that FPV had in vitro inhibitory activity against SARS-CoV-2 [69]. Cai et al. [90] compared the efficacy of FPV (35 patients) and LPV/r (45 patients) in the treatment of COVID-19 in an open-label, non-randomized controlled study, with FPV showing better clinical outcomes including improved chest CT and viral clearance. Similar superiority of FPV was demonstrated over umifenovir in a randomized (1:1) open-label controlled trial involving 240 patients in a multicenter setting [88]. Although the in vitro and limited clinical data are promising, more clinical evaluation and experience is desirable to ascertain the efficacy of FPV in COVID-19 treatment.

#### 3.2.4. Chloroquine and Hydroxychloroquine

Hydroxychloroquine (HCQ) and its parent compound, chloroquine (CQ), are aminoquinoline drugs with a long history of use in the treatment of protozoal infections, especially malaria and intestinal amebiasis. Both drugs have been used as disease-modifying agents for immunological disorders such as rheumatoid arthritis and lupus erythematosus. They were widely popularized for COVID-19 treatment, especially in the early days of the pandemic. Data from in vitro studies are mixed regarding the antiviral activity of CQ and HCQ. Earlier studies have demonstrated in vitro activities of the aminoquinolines against certain viral strains. In a study reported by Li et al., the replication of Epstein–Barr virus in infected cells was enhanced by CQ [91]. In other studies, CQ inhibited the vertical transmission of the Zika virus in infected mice [92]. The in vitro inhibition of Ebola virus replication by CQ did not translate into an in vivo improvement in infected guinea pigs [93,94]. This pattern was repeated with a promising inhibitory effect of CQ on the in vitro replication of dengue virus which did not translate to any significant clinical response in humans [95,96]. Reports of in vitro inhibition of SARS-CoV-2 replication by CQ and HCQ, besides the known immunomodulatory effects of these two drugs, led to speculations that the aminoquinolines may be an effective treatment for COVID-19 [97].

Apart from a few case reports and case series, most clinical studies have not found HCQ to be efficacious in the management of COVID-19. An initial clinical study of 100 patients claimed that chloroquine showed efficacy in the treatment of pneumonia associated with COVID-19 [98]. This study appeared isolated and was not corroborated by other clinical findings. Subsequent popularization of HCQ along with azithromycin, a staple macrolide antibiotic, for the treatment of COVID-19 generated a significant number of clinical reports with mixed inferences. In an open-label, non-randomized study reported by Gautret et al. [99], 20 hospitalized COVID-19 patients treated with HCQ (and azithromycin when necessary) had significantly reduced viral loads by day 6 of treatment compared to the untreated control group. However, in multiple randomized and/or control studies, HCQ was not associated with reduced incidence and duration of illness, a higher rate of negative conversion, or any other clinical measure of improvement [100,101,102,103]. Lack of supporting data has thus weakened the argument for the continued use of CQ/HCQ in the treatment of COVID-19, and has led to the withdrawal of the previously approved EUA [104].

### 3.3. Biologics, Immunomodulators and Other Supportive Treatments

#### 3.3.1. Anakinra

Anakinra is an immunosuppressive biologic that functions as an interleukin-1 receptor antagonist. The combination of ARDS and hyperinflammation-induced multi-organ failure caused by a cytokine storm is primarily responsible for COVID-19 deaths [105]. The biochemical and immunopathological changes observed in COVID-19 share similarities with immune-mediated disorders for which drugs such as anakinra have been used [106].

In several case studies, anakinra was found to be safe as an adjunct treatment for COVID-19. In patients with signs of inflammation, anakinra, when used with other treatments, is effective in alleviating symptoms, reducing the need for mechanical ventilation, and improving general clinical outcomes [107,108,109,110,111,112,113].

In an observational cohort study, for example, 120 hospitalized COVID-19 patients with associated hyperinflammation were monitored for the effect of a high-dose anakinra–methylprednisolone combination on 28-day survival [114]. The study, which compared 65 treated patients with 55 untreated historical controls, reported a significant reduction in mortality in the treatment group (13.9%) compared with controls (35.6%). Another retrospective cohort study analyzed outcomes in 29 patients managed with standard treatment (antiviral and HCQ) and anakinra, with 16 others managed with standard treatment only [115]. Use of anakinra was associated with better outcomes compared to the control group, as demonstrated by greater improved respiratory function (72% vs. 50%) and day 21 survival (90% vs. 56%). In both studies, anakinra was not associated with reductions in bacteremia. A third study reported no significant differences in the need and duration of mechanical ventilation use or length of ICU stay, although the study found a better clinical response with anakinra use [116]. Currently, there are several ongoing clinical trials related to the use of anakinra in the treatment of COVID-19. A recently published prospective, open-label, interventional study showed interesting and promising results [117]. A total of 45 patients in the intervention group received anakinra in addition to standard therapy compared to 24 historical controls. The use of anakinra was associated with a reduced need for mechanical ventilation, shorter duration of required oxygen therapy, and reduction in inflammatory biomarkers. The findings of this study presented some of the strongest evidence for the efficacy of anakinra in enhancing clinical outcomes in patients with severe COVID-19.

#### 3.3.2. Corticosteroids

Corticosteroids, such as methylprednisolone and dexamethasone, are standard treatments for inflammation-associated disorders. The severe inflammatory responses that often accompany SARS-CoV-2 infection have made the use of corticosteroids popular in the treatment of COVID-19. Expectedly, high-dose, short-term corticosteroid therapy in early respiratory distress has been associated with better prognosis [118]. Several clinical reports have demonstrated that the use of corticosteroids in severe COVID-19 provides multiple clinical benefits, including reductions in the need for and duration of invasive ventilation [119,120,121,122].

In a single-blind, randomized controlled trial with 34 patients in each arm to receive standard treatment with or without methylprednisolone, clinical improvement and survival was significantly better with the use of methylprednisolone (94.1% vs. 57.1%) [123]. However, these findings were not supported by the data from a double-blind placebo-controlled trial which randomized 416 patients to receive either methylprednisolone (194 patients) or a placebo (199) [124]. In the latter study, survival rate at day 28 was not different between the two groups, although a sub-analysis showed higher survival rate among patients who were over 60 years of age.

Similar results have been reported for dexamethasone. In a case series of 21 patients who had COVID-19 with pneumonia and worsening hypoxemia and were treated early with short-course dexamethasone, significant clinical improvements were reported, and none of the patients deteriorated to the point of requiring mechanical ventilation [125]. The RECOVERY trial, a controlled open-label study of 6425 hospitalized COVID-19 patients, provides the most elaborate study on the efficacy of dexamethasone [126]. The patients were randomized (1:2) to receive a 10-day course of dexamethasone or the usual care. Overall, 28-day mortality (22.9% vs. 25.7%) was only slightly lower in the dexamethasone group. However, sub-analysis showed that survival was better with dexamethasone for patients who were on mechanical ventilation (70.7% vs. 59.6%) and those receiving non-invasive oxygen support (76.7 vs. 73.8%). Cano et al. [127] performed a meta-analysis of the 73 available clinical studies covering the use of corticosteroids in 21,350 COVID-19 patients and concluded that mortality benefits were shown in patients who were severely ill. Thus, although corticosteroids do not have direct antiviral activity, their ability to suppress the deleterious acute inflammatory response associated with SARS-CoV-2 infection has made them clinically important in the management of COVID-19.

#### 3.3.3. Tocilizumab

Tocilizumab (TCZ) is an immunosuppressive monoclonal antibody used for the treatment of immune-mediated disorders. It is a potent antagonist of the interleukin-6 receptor. Similarly to anakinra and corticosteroids, TCZ has been employed as supportive therapy in COVID-19 to manage symptoms of hyper-inflammation and other immune responses. Several published case series and reports observed remarkable clinical responses following the use of TCZ in COVID-19 patients, including the clinical resolution of septic shock, reduction in the markers of inflammation, enhanced negative conversion, and reduced need for invasive mechanical ventilation [128,129,130,131,132,133,134,135,136,137,138,139].

#### 3.3.4. Interferons

Interferons (IFNs) are immunomodulators that can decrease the inflammatory response. They have been part of the standard treatment for hepatitis B and C viral infections. By enhancing the host’s immunological defense against the virus, interferons may act to suppress the replication of SARS-CoV-2 and provide anti-inflammatory effects as well. In particular, IFN-α2b, IFN-β1b, and IFN-β1a have been explored for therapeutic benefits in COVID-19 patients, although very few clinical studies have examined efficacy. In a cohort study, 7, 24, and 46 hospitalized patients received nebulized IFN-α2b, umifenovir, or an IFN-α2b–umifenovir combination (standard of care), respectively. The use of IFN-α2b alone or in combination was associated with significantly higher viral clearance and reduction in circulating biomarkers (IL-2 and CRP) of inflammation [140]. Similar positive results have been reported for IFN β-1a and IFN β-1b [141,142,143]. It is anticipated that INFs will continue to play at least supportive roles in COVID-19 treatment while more clinical data are being collected.

#### 3.3.5. Tissue Plasminogen Activator

Tissue plasminogen activator (TPA) is a thrombolytic agent that has been used as supportive treatment for severely hypoxic COVID-19 patients. It is hypothesized that TPA administration may help patients who are experiencing thrombus formation by immediately lysing and diffusing the thrombus, thereby improving oxygen levels [144]. Apart from the significant bleeding risk anticipated from this biochemical mechanism, convincing therapeutic benefits have not been reported with the use of TPA in COVID-19 patients. In the few published case series, only temporary clinical improvement was observed with TPA use [145,146]. As our understanding of COVID-19 disease progression improves, better treatment options may relegate TPA to only occasional symptom-dependent support therapy.

#### 3.3.6. Intravenous Immunoglobulin

Intravenous immunoglobulin (IVIG) is a blood product which is used to supplement antibodies produced by the patient to enhance the immunological suppression of pathogens. In a published report of the first COVID-19 case in Bhutan, LeVine et al. [147] attributed a dramatic clinical response in an immunocompromised 76-year-old patient to a three-day course of IVIG. The patient’s condition had deteriorated despite treatment with oseltamivir, ceftriaxone, and doxycycline. Similar dramatic responses have been attributed to IVIG [148,149,150]. While questions on dosing and safety will need to be addressed through further studies, IVIG may continue to offer a treatment option for refractory cases of COVID-19.

#### 3.3.7. Nafamostat

Nafamostat is a proteolytic enzyme inhibitor approved and marketed in Japan for the treatment of pancreatitis and coagulation disorders. In vitro activity of nafamostat against the MERS coronavirus has been reported previously [151,152]. Some anecdotal reports have associated nafamostat with enhanced clinical outcomes, especially when used along with other treatments [153]. According to clinical reports on eleven COVID-19 cases, nafamostat has been reported to reduce viral replication and enhance recovery when used in combination with FPV [154]. However, while nafamostat may be a potential treatment, little clinical experience exists for its use in COVID-19 therapy.

The summary of the clinical experience with repurposed and off-label-use drugs for the treatment of COVID-19 is provided in Table 2.

#### 3.3.8. Vaccines

The Pfizer/BioNTech (BNT162b2) and Moderna (mRNA-1273) COVID-19 vaccines, both of which contain nucleoside-modified RNAs encoding the SARS-CoV-2 spike protein, were the first to be approved by the U.S. FDA under Emergency Use Authorization in December 2020. Following successful phase 3 clinical trials, the vaccines’ preliminary efficacy (95%) and safety data supported a two-dose regimen [172]. The Oxford/AstraZeneca (AZD1222) vaccine, developed as a viral vector vaccine, was first approved in Europe for a two-dose regimen, having demonstrated an initial efficacy of 82.4%. Similar adenovirus vector vaccines that have been developed and approved for public use include the Sputnik (Russia), Johnson & Johnson (U.S. and Europe), and Convidecia (China). Other COVID-19 vaccines have been developed from inactivated virus and included Sinopharm (China), CoronaVac (China), covaxin (India), CoviVac (Russia) and QazCovid-in (Kazakhstan). EpiVacCorona (China) and RBD-Dimer (ZF-2001) (China) are two other vaccines made from viral protein subunits. All of these vaccines have shown some degree of efficacy and have been approved in different countries/regions of the world. The efficacy of these vaccines against emerging SARS-CoV-2 variants is currently not clear. The availability of new data continues to inform regulatory decisions, and most recently (May 2021), the U.S. FDA approved the use of the Pfizer/BioNTech (BNT162b2) vaccine in adolescents aged 12 years and older. Information on global vaccine listings are being maintained and updated by the WHO [173].

#### 3.3.9. Adjunctive and Supplementary Medicines

The role of vitamins and herbal products as complementary therapies in the management of COVID-19 has been the subject of several recent publications [174,175]. Vitamins serve as antioxidants and enhance immunity. They may also help to accelerate a patient’s recovery from the multi-dimensional symptomatology of COVID-19.

Multiple case reports and case series suggest that high-dose vitamin C may enhance the recovery from COVID-19 [176,177,178]. A retrospective study of 76 patients reported that the use of vitamin C in COVID-19 patients was associated with modest clinical benefits, including reductions in 28-day mortality and improvements in oxygen support status [179]. However, more elaborate clinical studies did not demonstrate therapeutic benefits of high-dose vitamin C in patients with COVID-19 [180,181,182,183]. Similarly, vitamin D supplementation may be of potential benefit in ameliorating the effects of COVID-19, but there are currently no clinical studies to support this suggestion [184].

There are anecdotal reports on the therapeutic efficacy of herbal medicines. Traditional Chinese medicine (TCM) is perhaps the most studied in this regard. There are several ongoing clinical trials examining the therapeutic effects of some of these herbal products. Beyond traditional use, none of these products have been officially approved for in-patient treatment of COVID-19. A detailed review of herbal therapies in COVID-19 treatment is beyond the scope of the current paper.

## 4. Discussion

The response of the global scientific community to the COVID-19 pandemic has been impressive. The use of repurposed drugs for clinical management of the disease, the testing of new drugs in clinical trials, and the successful development of several efficacious vaccines within one year are the results of a monumental worldwide effort to restrict SARS-CoV-2 infection. While the experimental development and regulatory approval of current drugs and vaccines have been rapid and fast-tracked, sufficient clinical data have been generated to warrant their use in the prevention and management of COVID-19. However, the long-term effects of drugs can only be assessed after several years of clinical experience. The efficacy and safety of current COVID-19 therapeutics should continue to be monitored as part of a rigorous pharmacovigilance process.

Several repurposed and supportive drugs have shown mixed data on efficacy and safety. Interpreting the published studies can be challenging due to multiple confounding factors, of which the most prominent factor is the lack of effective standard COVID-19 control treatment. The use of multiple regimens as standard control therapy blurs the line of the attribution of efficacy to the test drugs. In addition, the sample sizes in most of the studies were too small, and the consequent small statistical power limits the inferences that can be drawn from the results. The presence of comorbidities and the severity of COVID-19 in many of the studied patients also complicates understanding the pathophysiology of the disease as well as the pharmacodynamic response.

Although the advent of vaccines will continue to reduce the spread and the global burden of COVID-19, the disease may not be totally eradicated due to a combination of viral mutation and refusal of individuals to be vaccinated. Therefore, a reliance on pharmacotherapy in COVID-19 management will likely continue. It is evident that targeted antiviral treatment and multiple supportive therapies, including immunomodulation and antibody supplementation, will continue to play significant pharmacological roles in the treatment of COVID-19. Prudent use of these available drugs will hopefully continue to benefit patients while more effective therapeutics are being developed.

## Figures and Tables

**Table 1:** Summary of clinical findings on the effectiveness of current therapeutics for COVID-19.

Authors	Study Design	Description	Findings
**Remdesivir**			
Wang et al., 2020 [31]	Randomized, double-blind, placebo-controlled, multicenter clinical trial	Total of 237 patients enrolled (158 and 79 randomized to remdesivir and placebo group, respectively) with concurrent use of other antivirals and corticosteroids	Although patients taking remdesivir had faster clinical improvement, differences in time to clinical improvement in the two groups were not statistically significant.
Antinori et al., 2020 [32]	Prospective open-label study	Total of 35 critically ill COVID-19 patients (18 ICU, 17 IDW) enrolled	At day 28, 6 and 14 patients were discharged from the ICU and IDW, respectively. Remdesivir was believed to enhance clinical improvement.
Grein et al., 2020 [33]	Case series	53 patients received remdesivir on compassionate use grounds.	At follow-up (median of 18 days), significant clinical improvement was observed in 36 patients, including 17 out of 30 extubated, and 25 patients discharged.
Maldarelli et al., 2020 [34]	Case report	39-year-old pregnant woman in the ICU for COVID-19 was given remdesivir on compassionate use grounds.	Patient discharged on day 9 after completing 8 out of the planned 10-day remdesivir therapy. Emergency delivery was not required.
Dubert et al., 2020 [35]	Case series	First cases of five (5) patients hospitalized with COVID-19 and treated with remdesivir based on compassionate use in France.	Despite significant decrease in viral load in all patients, remdesivir use had to be interrupted in 4 patients (2 because of elevated liver enzymes and 2 because of nephrotoxicity). Two patients died.
Helleberg et al., 2021 [36]	Case report	Immunocompromised patient in his 50s treated for COVID-19 with two 10-day courses of remdesivir at 24 and 45 days after onset of symptoms. No adjunctive corticosteroid was used.	Symptoms improved. Patient tested negative by day 38 and was discharged by day 65.
Beigel et al., 2020 [37]	Randomized, double-blind, placebo-controlled trial	Total of 1062 patients randomized with 541 receiving remdesivir (200 mg day 1, and 100 mg daily for 7 days) and 521 receiving placebos.	Median recovery time with remdesivir was 10 days compared to 15 days in placebo group. Incidence of serious adverse event was lower in the remdesivir group.
Goldman et al., 2020 [38]	Randomized, open-label, phase 3 trial	Total of 397 COVID-19 patients randomized into 200 and 197 receiving intravenous remdesivir for 5 or 10 days, respectively (200 mg day 1, then 100 mg daily subsequently).	Clinical improvement by day 14 was similar in both groups.
**Casirivimab/Imdevimab (Regeneron)**			
Weinreich et al., 2020 [15]	Double-blind, phases 1–3	Total of 275 patients randomized equally into 3 groups: placebo and 2.4 g or 8.0 g of casirivimab/imdevimab combination.	Safety profiles was similar in tests and placebo. Significant reduction in viral load was associated with the drug compared to the placebo.
**Convalescent Plasma**			
Jafari et al., 2020 [39]	Case report	26-year-old COVID-19 patient pregnant with twins delivered via caesarean section, and then was treated with meropenem, azithromycin, and hydroxychloroquine. CP was given on day 6 of hospitalization.	Significant clinical response was observed, and patient was discharged after 2 weeks.
Figlerowicz et al., 2020 [40]	Case report	6-year-old diagnosed with severe COVID-19 whose treatment did not respond to antiviral and immunomodulatory drugs.	Viral elimination after the initiation of CP
Im et al., 2020 [41]	Case report	68-year-old with severe COVID-19 treated with hydroxychloroquine and antiviral drugs; later transfused with CP.	Patient was discharged after 12 days. Showed significant improvement within 3 days after CP infusion.
Xu et al., 2020 [42]	Case report	Critically ill 65-yer-old patient with COVID-19 treated with 2 rounds of CP infusion and 7-day course of oral HCQ.	Patient remained positive after 11 days of treatment with minimal symptom improvement. Response to the combination was not optimal.
Ye et al., 2020 [43]	Case series	Six COVID-19 patients received between 1 and 3 cycles of CP infusion.	Significant symptom resolution and viral cure in all patients with no major side effects: 5 discharged; 1 stable and under clinical monitoring.
Abdullah et al., 2020 [44]	Case series	Two patients with severe COVID-19 that was refractory to antiviral and supportive treatment.	Initiation of CP was accompanied by significant improvement with resultant cure and negative virology tests
Duan et al., 2020 [45]	Case series	Ten patients with severe cases who received 250 mL single-dose infusion of CP.	Rapid clinical improvements and viral cure within 7 days.
Fung et al., 2020 [46]	Case series	Four immunocompromised patients (3 transplant recipients, and one with chronic myelogenous leukemia) who contracted COVID-19 and were treated with CP.	Clinical improvement in all 4 patients with 2 fully recovered and the other 2 discharged to skilled nursing facilities.
Wang et al., 2020 [47]	Case series	Five patients with severe COVID-19 associated with severe respiratory failure who required mechanical ventilation and were treated with CP.	Two patients were cured, while 3 died due to multiple organ failure. CP was initiated late (median time from symptom onset was 37 days.
Olivares-Gazca et al., 2020 [48]	Case series	Ten patients with severe COVID-19 were treated with CP and adjunctive therapies	Significant improvement in the measures of organ damage in all patients; improved chest X-ray and CT scans in 7 and 6 patients, respectively; 3 out of 5 removed from mechanical ventilation, 6 cured and discharged, and 2 died.
Shen et al., 2020 [49]	Case series	Five patients who developed critical illness including acute respiratory distress from COVID-19. All were on mechanical ventilation and received antiviral and corticosteroid therapy while being treated with CP.	Symptoms improved significantly after CP infusion. By day 37, 3 patients had been discharged home while the other 2 were in stable condition.
Ahn et al., 2020 [50]	Case series	Two severely ill patients with COVID-19 whose conditions did not improve by mechanical intubation, antiviral and supportive therapies. Both were treated with CP.	Symptoms improved. Patients were extubated and tested negative (after 20 and 26 days) with one discharged and the other stable.
Zeng et al., 2020 [51]	Case series	Six severely ill patients with respiratory failure due to COVID-19 were treated with CP (21.5 median days after testing positive).	All 6 had viral clearance (testing negative within 3 days after CP infusion). CP did not reduce mortality (5 patients died) probably because of late initiation, and patients were critically ill.
Salazar et al., 2020 [52]	Case series	Total of 25 patients with severe COVID-19 illness enrolled. Patients were transfused with CP with outcomes of safety and clinical status 14 days post-infusion. CP administered in addition to antiviral and other supportive treatments.	No adverse event reported in any of the patients; 19 patients showed clinical improvements by day 14, and 11 were discharged. At the time of publication, 20 of the 25 patients had been discharged.
Li et al., 2020 [53]	Open-label, multicenter, randomized clinical trial	A total of 103 COVID-19 patients with severe or life-threatening symptoms enrolled and randomized to evaluate the efficacy of add-on CP to standard therapy.	CP did not cause any significant difference in 28-day mortality compared to standard therapy. Differences in time to discharge were not significant. Study was terminated early, without reaching the planned 200 recruits.

Abbreviations: CP—convalescent plasma; ICU—intensive care unit; IDW—infectious disease ward; HCQ—Hydroxychloroquine

**Table 2:** Clinical experience with repurposed and off-label-use drugs for the treatment of COVID-19.

Drug and Study Type	Description	Findings	References
LPV/r, case report	Index (54-year-old) patient in a Korean hospital treated with LPV/r 10 days after disease onset.	Significant decrease in viral load after LPV/r administration. LPV might have played a role.	[155]
LPV/r, case report	65-year-old HIV/AIDS patient being treated with LPV/r who contracted SARS-CoV-2. Support treatment was added.	Patient improved and was discharged after 34 days. LPV/r was thought to play a role in recovery.	[156]
LPV/r, case report	35-year-old patient treated with LPV/r for 10 days, along with supportive therapies.	Virological cure was confirmed, and patient was discharged.	[157]
LPV/r, case series	5 cases of COVID-19 with 2 patients treated with LPV/r while 3 patients served as controls for the analysis.	The rate and duration of SARS CoV-2 shedding was not different with or without LPV/r.	[158]
LPV/r, case series	3 patients received LPV/r for 3, 10, and 12 days, several days after the onset of illness.	All patients recovered, tested negative, and were discharged.	[159]
LPV/r, azithromycin, HCQ; case series	Two cases of immunosuppressed patients who were managed with drug combinations.	Despite being recipients of kidney transplants, both patients recovered after drug treatments.	[160]
LPV/r, AZM and HCQ combination; case report	Severe COVID-19 case in a 41-year-old who was treated with the combination therapy.	Most multi-organ symptoms resolved within 10 days and patient was discharged after 2 weeks of hospitalization.	[161]
LPV/r, controlled open-label	47 hospitalized COVID-19 patients were grouped to either receiving or not receiving LPV/r in addition to their adjuvant therapies.	LPV/r was associated with faster clinical response and a shorter disease course.	[77]
LPV/r, randomized, controlled, open-label trial	199 hospitalized severe COVID-19 patients were randomized to receive either a 14-day course of LPV/ritonavir in addition to standard care or standard care alone.	LPV/r neither shortened the time to clinical improvement nor reduced mortality at 28 days. Virological response was similar in both groups.	[78]
LPV/r, HCQ, and interferon β-1b combination; case series	5 patients with severe cases of COVID-19 treated with the combination therapy, in addition to corticosteroids for associated inflammation.	Clinical improvement and resolution of symptoms were observed. All 5 patients were discharged.	[162]
LPV/r, ribavirin and interferon combination; open-label, randomized, phase 2 trial	127 patients were randomized 86:41 to receive a 14-day course of either the combination or the control LPV/r only.	The combination therapy was associated with shorter duration of viral shedding and hospitalization.	[163]
Umifenovir, case control	Retrospective analysis of 50 cases of COVID-19 patients treated with LPV/r (34 cases) or umifenovir (16 cases).	Disease progression was halted in both groups. Viral cure at day 14 was 100% and 56%in umifenovir and LPV/r groups, respectively.	[84]
Umifenovir, case control	62 hospitalized COVID-19 patients were analyzed based on whether they received adjuvant therapy alone (20, control) or with umifenovir (test, 42).	The use of umifenovir was associated with a shorter course of disease and reduced duration of hospitalization.	[85]
Umifenovir; retrospective cohort study	Analysis of patients treated with umifenovir-LPV/r combination (16 patients) compared to LPV/r only (17 patients).	By day 7 of treatment, negative conversion occurred in 75% of the patients in the combination group compared to 35% in LPV/r group. There was better chest CT scan improvement with the combination.	[83]
Umifenovir, case control	A retrospective analysis of 81 hospitalized patients treated for COVID-19 (45 umifenovir and 36 control).	Clinical outcomes were not better with umifenovir.	[87]
Umifenovir, randomized controlled trial	86 patients randomized as follows: 34 LPV/r, 35 to umifenovir, and 17 control, no antiviral medication.	Viral cure rate and clinical responses were not significantly different in the groups.	[86]
Umifenovir; randomized open-label controlled trial	240 patients were randomized (1:1) in a multicenter study to receive conventional COVID-19 therapy plus either umifenovir or favipiravir.	Clinical recovery by day 7 was not significantly different between the groups, but umifenovir was inferior to favipiravir in shortening the duration of symptoms.	[88]
HCQ; case report	60-year-old who was taking HCQ for 6 months for Sjogren’s syndrome contracted SARS-CoV-2 and had illness.	Chronic use of HCQ did not prevent COVID-19.	[164]
HCQ, tocilizumab; case report	61-year-old immunocompromised transplant recipient diagnosed with COVID-19 and treated with HCQ and tocilizumab.	Patient experienced significant clinical improvement and was discharged 13 days after diagnosis.	[165]
HCQ, AZM; case report	74-year-old COVID-19 patient with significant comorbidities was managed in the ICU with HCQ and AZM.	Patient recorded significant clinical improvement and was extubated by day 5 and moved to the floor.	[166]
HCQ, AZM; open-label	Analysis of 1376 hospitalized patients treated with one or a combination of HCQ and AZM	AZM alone was associated with reduced mortality compared to no treatment. HCQ did not affect mortality.	[167]
HCQ; case series	3 cases of chronic HCQ users who contracted SARS-CoV-2 and had serious symptoms.	Chronic HCQ use did not prevent COVID-19.	[168]
HCQ observational study	Analysis of 1446 patients to establish association between HCQ use and intubation or death.	HCQ did not reduce or increase the need for intubation or incidence of death.	[100]
HCQ, AZM; Open label, non-randomized study	20 hospitalized COVID-19 patients treated with HCQ (and AZM when necessary) with outcome of viral load suppression compared to untreated patients.	HCQ and AZM were associated with significantly reduced viral load by day 6 of treatment compared to untreated control.	[99]
HCQ; randomized, double-blind, placebo-controlled trial	821 participants who had been exposed to COVID-19 but were asymptomatic were randomized to receive either HCQ or placebo for post-exposure prophylaxis.	HCQ did not reduce the incidence of illness, but rather was associated with a higher incidence of side effects.	[101]
HCQ; randomized open-label, multicenter, controlled trial	150 hospitalized COVID-19 patients randomized (1:1) to receive HCQ or not, in addition to standard care.	The use of HCQ was not associated with a higher rate of negative conversion of SARS-CoV-2. HCQ was associated with higher incidence of side effects	[102]
CQ and tocilizumab, case report	63-year-old hospitalized for COVID-19 and treated with a 7-day course of CQ and single IV tocilizumab.	Patient experienced significant clinical improvement, recovered, and was discharged.	[169]
CQ, randomized phase II trial	Patients were enrolled in a study to compare the efficacy and safety of high-dose (81 patients) vs. low-dose (40 patients) CQ as adjunct therapy for severe COVID-19.	High CQ dose was associated with higher incidence of side effects. High-dose CQ did not have a better effect on viral load than low CQ dose.	[103]
AZM; open-label, randomized multicenter	Study of randomized 397 hospitalized patients with severe COVID-19 to either receive (214) or not (183) receive AZM in addition to standard treatment which included HCQ.	AZM was not associated with significant clinical improvement.	[170]
TCZ, case report	42-year-old cancer patient who had respiratory failure as a complication of COVID-19 despite treatment with LPV/r. He was treated with two infusions of TCZ.	Patient experienced rapid clinical improvement and was fully discontinued on oxygen 5 days after TCZ infusions. Patient later fully recovered.	[128]
TCZ; case report	Critically ill 57-year-old with COVID-19 who was refractory to standard treatment and treated with TCZ to inhibit cytokine storm.	Significant and progressive clinical response was observed in response to TCZ.	[129]
TCZ; case report	54-year-old with severe respiratory symptoms from COVID-19 who did not respond to antiviral drugs and was infused with TCZ.	Remarkable clinical improvement was observed only 4 days after TCZ administration.	[130]
TCZ; case report	36-year-old severe COVID-19 patient whose symptoms did not improve with HCQ and antiviral drugs. A single-dose TCZ was infused.	Progressive improvement was observed after TZC use, with subsequent negative conversion and recovery.	[131]
TCZ; case report	46-year-old patient in ICU whose COVID-19 illness was refractory to HCQ, and other supportive therapy was treated with TCZ.	Patient experienced remarkable recovery and was discharged to home 5 days after TCZ use.	[133]
TCZ; case series	5 critically ill COVID-19 patients whose illness was refractory to standardized treatment.	Marked clinical improvement was observed in all patients except one. Recovery and negative conversion were reported.	[134]
TCZ; case series	Two patients whose COVID-19 was complicated by cytokine release syndrome were treated with TCZ.	Progression to secondary hemophagocytic lymphohistiocytosis was observed in both patients, with viral myocarditis in one, despite the treatment.	[135]
TCZ, case series	A retrospective analysis of 15 COVID-19 patients treated with TCZ with or without adjunct corticosteroids.	TCZ was associated with significant clinical improvement and the amelioration of cytokine storms in COVID-19 patients.	[136]
TCZ, case series	A retrospective analysis of 5 patients with severe COVID-19 illness requiring ICU admissions who were treated with TCZ.	All patients had significant improvement and were discharged from ICU after 13–26 days, with 2 discharged home.	[137]
TCZ; case series	3 patients admitted and treated with HCQ and AZT with no significant clinical improvement. All 3 received doses of TCZ.	Patients had sufficient clinical improvement to avoid intubation, and ultimately recovered.	[138]
TCZ; case series	2 patients whose symptoms worsened after treatment with HCQ, AZM and other supportive therapies were administered with TCZ.	Drastic improvement in respiratory symptoms and markers of inflammation were observed following TCZ use. Both patients recovered and were discharged.	[139]
TCZ, case series	2 patients with severe COVID-19 illness refractory to standard therapy including HCQ, AZM, and antiviral drugs	Remarkable clinical resolution of septic shock and respiratory symptoms within 72 h.	[171]
TCZ, non-controlled, prospective	42 patients with severe COVID-19 were treated with single 400 mg TCZ infusion. Primary outcome was a reduction in the need for invasive mechanical ventilation and death.	Only 6 patients required invasive mechanical ventilation. Total of 7 patients died by day 8.	[132]
IFN-α2b; prospective cohort study	Hospitalized patients were treated with nebulized IFN-α2b (*n* = 7), umifenovir (*n* = 24), or IFN-α2b–umifenovir combination (standard of care; *n* = 46)	The use of IFN-α2b alone or in combination was associated with significantly higher viral clearance and reduction in circulating biomarkers (IL-2 and CRP) of inflammation.	[140]
IFN-β-1a; prospective non-controlled study	Observation and analysis of 20 patients treated with IFN-β-1a in addition to conventional treatment (HCQ and LPV/r).	Significant clinical response including viral clearance and symptom relief. Recovery after 14 days, with no serious adverse events in any patient.	[141]
IFN-β-1a; randomized controlled trial	Patients (*n* = 44) were treated with IFN-β-1a in addition to standard treatment and compared with controls (39) who received standard treatment only.	Mortality at day 28 was significantly lower in patients treated with IFN-β-1a compared to control (19% vs. 43.6%). IFN-β-1a did not shorten the time to clinical response.	[142]
IFN-β-1b, randomized, open-label trial	Patients received IFN-β-1b in addition to standard treatment (*n* = 33) and were compared to controls who received only the standard treatment (*n* = 33).	IFN-β-1b shortened the time to clinical improvement (9 vs. 11 days); enhanced recovery and 14-day discharge (78.79% vs. 54.55%); reduced ICU admission (42.42% vs. 66.66%) and all-cause 28-day mortality (6.06% vs. 18.18%).	[143]

Abbreviations: AZM—azithromycin; CQ—chloroquine; HCQ—hydroxychloroquine; INF—interferon; LPV/r—ritonavir-boosted lopinavir; TCZ—Tocilizumab.

## Data Availability

Not applicable.
